# miR-31-5p as a Potential Circulating Biomarker and Tracer of Clinical Improvement for Chronic Inflammatory Demyelinating Polyneuropathy

**DOI:** 10.1155/2023/2305163

**Published:** 2023-04-10

**Authors:** Edyta Dziadkowiak, Dagmara Baczyńska, Małgorzata Wieczorek, Mateusz Olbromski, Helena Moreira, Monika Mrozowska, Sławomir Budrewicz, Piotr Dzięgiel, Ewa Barg, Magdalena Koszewicz

**Affiliations:** ^1^Department of Neurology, Wroclaw Medical University, Borowska 213, 50-556 Wroclaw, Poland; ^2^Department of Molecular and Cellular Biology, Wroclaw Medical University, Borowska 211A, Wroclaw, Poland; ^3^Faculty of Earth Sciences and Environmental Management, University of Wroclaw, Uniwersytecki 1, 50-137 Wroclaw, Poland; ^4^Department of Histology and Embryology, Wroclaw Medical University, ul. Chałubinskiego 6a, 50-368 Wroclaw, Poland; ^5^Department of Basic Medical Sciences, Wroclaw Medical University, Borowska 211, 50-556 Wroclaw, Poland

## Abstract

**Background:**

MicroRNAs are endogenous, small noncoding RNA molecules that play a pivotal role in the regulation of gene expression. MicroRNAs are involved in many biological processes such as proliferation, cell differentiation, neovascularization, and apoptosis. Studies on microRNA expression may contribute to a better understanding of the pathomechanism of chronic inflammatory demyelinating polyneuropathy (CIDP) and consequently enable the development of new therapeutic measures using antisense miRNAs (antagomirs). In this study, we evaluated the level of miR-31-5p in the serum of patients with CIDP and its correlation with the miR-31-5p level and clinical presentation and electrophysiological and biochemical parameters.

**Methods:**

The study group consisted of 48 patients, mean age 61.60 ± 11.76, who fulfilled the diagnostic criteria of a typical variant of CIDP. The expression of miR-31-5p in patient serum probes was investigated by droplet digital PCR. The results were correlated with neurophysiological findings and the patient's clinical and biochemical parameters.

**Results:**

The mean copy number of miRNA-31 in 100 *μ*l serum was 1288.64 ± 2001.02 in the CIDP group of patients, while in the control group, it was 3743.09 ± 4026.90. There was a significant positive correlation (0.426) between IgIV treatment duration and miR-31-5p expression. Patients without IgIV treatment showed significantly lower levels of miR-31 compared to the treated group (259.44 ± 304.02 vs. 1559.48 ± 2168.45; *p* = 0.002). The group of patients with body weight > 80 kg showed statistically significantly lower levels of miRNA-31-5p than the patients with lower body weight (934.37 ± 1739.66 vs. 1784.62 ± 2271.62, respectively; *p* = 0.014). Similarly, the patients with elevated cerebrospinal fluid (CSF) protein levels had significantly higher miRNA-31-5p expression than those with normal protein levels (1393.93 ± 1932.27 vs. 987.38 ± 2364.10, respectively; *p* = 0.044).

**Conclusion:**

The results may support the hypothesis that miR-31-5p is strongly involved in the autoimmune process in CIDP. The positive correlation between higher miR-31-5p levels and duration of IVIg treatment may be an additional factor explaining the efficacy of prolonged IVIg therapy in CIDP.

## 1. Introduction

MicroRNAs (miRNAs) are a family of single-stranded, noncoding gene expression, endogenous regulatory molecules formed from double-stranded precursors. miRNAs are a group of molecules about 21-23 nucleotides in length that posttranscriptionally regulate gene expression and thus contribute to the modulation of numerous complex and disease-relevant cellular processes, including cell proliferation, cell motility, cell cycle control, neovascularization, apoptosis, and stress response. Their role depends mainly on their complementarity to the 3′UTR regions of targeted miRNAs. The importance of these molecules is demonstrated by the fact that more than one-third of protein-coding genes in human cells are regulated by miRNAs. It was estimated that genes encoding miRNAs account for 1-5% of all genes in humans and animals [1–3]. Previous studies had reported that miR-31-5p plays an essential role in tumor suppression in hepatocellular carcinoma by regulating the cell cycle and epithelia-mesenchymal transition. In addition, the altered expression of miR-31-5p was also confirmed in other tumors such as esophageal squamous cell carcinoma, gastric cancer, colorectal cancer, breast cancer, and cervical cancer [4–6].

On the other hand, together with other miRNAs such as miR-1, miR-133a, miR-133b, and miR-206, miR-31 belongs to dystromirs (Dystromirs as Serum Biomarkers for Monitoring the Disease Severity in Duchenne Muscular Dystrophy) due to the specific expression in muscle cells and roles in skeletal muscle maintenance and regeneration [7]. It has been shown that miR-31 is involved in muscle proliferation and differentiation, as it increases myoblast transformation in myotubes [8]. Furthermore, miR-31 modulates dystrophin expression by targeting the 3′UTR of the dystrophin transcript and repressing its translation. Therefore, miR-31 has been suggested as a suitable target for improving dystrophin recovery in exon skipping therapy in Duchenne muscular dystrophy [7, 9].

The level and effect of miR-31 thus vary by the type of the autoimmune disease [10–12]. miR-31 expression increases in the mice T cells in experimental autoimmune encephalomyelitis (EAE) and keratinocytes of patients with psoriasis, and its activation is associated with exacerbation of both diseases [12–14]. In contrast, patients with systemic lupus erythematosus have decreased expression of miR-31 in T cells, where it plays protective roles [15]. Moreover, miR-31 may also modulate the immune response of neutrophils [12].

The immune system homeostasis is mediated by regulatory T (Treg) cells [10]. miR-31 regulates Treg cells through several mechanisms, including suppression of FOXP3 (forkhead box P3) protein involved in Treg cell differentiation. Furthermore, miR-31 can repress the generation of peripherally derived Treg cells [11], which differentiate into secondary lymphoid organs and tissues to control autoimmune responses under specific inflammatory conditions [12]. Zhang et al. [13] identified a potential FOXP3-binding site within the promoter region of the gene encoding murine miR-31, suggesting that FOXP3 may directly target miR-31. FOXP3+ Treg cells are critical in maintaining immune tolerance and homeostasis of the immune system. The molecular mechanisms underlying the stability, plasticity, and functional activity of Treg cells have been much studied in recent years. Therefore, identifying the molecular mechanisms by which FOXP3 and miR-31 regulate each other and identifying the other downstream target genes in this regulatory network can assist in the development of novel treatments for autoimmune diseases [16, 17].

Chronic inflammatory demyelinating polyneuropathy (CIDP) is an acquired autoimmune neuropathy in which nerve damage occurs by both cellular and humoral mechanisms. The incidence rates varied between 0.15 and 0.70 cases per 100,000 persons per year. This disease occurs in all age groups, but its prevalence increases with age and is more common in men than in women. The median disease duration to diagnosis was ten months (range, 2-64) [18–20].

While various antibodies have been identified, including IgG4 classes directed against proteins of the Ranvier node and the nodal area, the diagnosis of CIDP is still based on clinical and electrophysiological criteria [18, 21, 22]. There is no specific biomarker characteristic for CIDP, which seems to be much-needed. Research on miRNAs appears to be helpful in the search for diagnostic markers and may contribute to the development of potential treatment. The incorrect expression of particular miRNAs may result from genome changes or abnormalities in their biogenesis or may be related to epigenetic factors regulating gene expression. Not only are the structurally determined changes in miRNA expression levels significant, but also their epigenetic regulation. Modulation of miRNA expression by changes in the methylation level of their genes will probably be a future target for new therapies in CIDP.

The aim of this study was to evaluate the expression of miR-31-5p in serum in CIDP patients with the assessment of the correlation between miR-31-5p level and clinical presentation and electrophysiological and biochemical parameters. Electrodiagnostics (nerve conduction studies) are recommended to confirm the clinical diagnosis of typical CIDP, so special attention was paid to the analysis of the correlation of electrophysiological parameters with miR-31-5p levels. In addition, we examined whether diabetes mellitus could influence the level of the miRNA-31 level. CSF analysis should be considered to exclude diagnoses other than CIDP; in particular, elevated protein levels in CSF should be interpreted with caution in the case of diabetes mellitus comorbidity. We also analyzed the relationship between IVIg treatment duration and miRNA expression.

## 2. Materials and Methods

The study group consisted of 48 patients (female: 8, male: 40), who fulfilled the CIDP diagnostic criteria according to the European Academy of Neurology/Peripheral Nerve Society guideline [18]. The mean age was 61.60 ± 11.76 years. A plasma exchange (5 exchanges over 2 weeks) procedure was performed in 23 patients with CIDP. Chronic immunosuppressive treatment was used in 43 patients, including corticosteroids (prednisolone) in 20 patients, and immunosuppressive drugs in 13 patients (mycophenolate mofetil in 9 patients, azathioprinum in 3 patients, and ciclosporin in 1 patient). In addition, 17 patients had diabetes mellitus type 2, including a history of plasma exchange for the immediate treatment of CIDP, while 9 patients received chronic immunosuppressive therapy (azathioprinum in 4 patients and mycophenolate mofetil in 5 patients). Hypertension was recognized in 23 patients. Also, 23 patients had degenerative changes of the lumbosacral vertebral column.

Patients with variants of CIDP (distal, multifocal, focal, motor, and sensory), chronic immune sensory polyradiculopathy (CISP), multifocal motor neuropathy (MMN), mononeuritis multiplex, hereditary demyelinating neuropathy, chronic ataxic neuropathy with disialosyl antibodies (CANDA) and chronic ataxic neuropathy, ophthalmoplegia, IgM paraproteinaemia, cold agglutinin and disialosyl antibodies (CANOMAD), cerebellar ataxia, neuropathy, vestibular areflexia syndrome (CANVAS), and POEMS syndrome were excluded from this study. Patients with IgG or IgA monoclonal gammopathy of undetermined significance and IgM monoclonal gammopathy without antibodies to MAG were also excluded. HIV infection, other immunological conditions, and malignancy were also excluded. Amyloidosis, liver and kidney damage, multiple myeloma and osteosclerotic myeloma, venous insufficiency and chronic limb ischaemia, myopathy and neuromuscular junction disease, dorsal column lesions (such as syphilis, paraneoplastic diseases, and copper deficiency) and toxic neuropathies (e.g., due to chemotherapy and vitamin B6 poisoning), and peripheral nerve tumors were excluded.

Vitamin B12 levels in all patients were normal. All patients were negative for tumor markers, antibodies against neuronal antigens, and antibodies characteristic for autoimmune disorders, including connective tissue diseases. All patients showed a good response to the treatment, IVIg in particular.

The control group consisted of 13 subjects. The control group was matched for sex, age, comorbidities, IBM index, and smoking.

### 2.1. Ethical Standards

All subjects in the patient and control groups were informed in detail about the purpose and procedure of the study and gave written informed consent to participate in the study. The authors had a positive opinion of the Bioethics Committee of the Medical University of Wrocław No. KB-719/2021 on conducting this study.

### 2.2. Clinical and Biochemical Evaluation

Clinical improvement was defined according to the following scales: INCAT disability scale (the significance was established as ≥1 point), Medical Research Council (MRC) sum score (as ≥2 to 4 points), and grip strength measured by hand dynamometer (+ more than 10% of the output value). The Inflammatory Neuropathy Cause And Treatment (INCAT) disability scale was first used in a clinical trial comparing the efficacy and safety of intravenous immunoglobulin with oral prednisone in patients with CIDP [23]. The scale is a measure of activity limitation by assessing upper and lower limb dysfunction. The INCAT score is inversely related to function, where 0 indicates no functional impairment and 10 indicates an inability to perform any purposeful movement of the limbs [24].

Serum microRNA-31 copy number; immunoglobulin IgG, IgM, IgA and levels; and creatine kinase (CK) activity were estimated. In addition, pleocytosis and protein levels were determined in cerebrospinal fluid.

Correlations between miRNA-31 expression and disease duration, clinical parameters (upper limb, lower limb, total INCAT), IgIV treatment time, body weight, and selected biochemical parameters (IgG, IgM, IgA, CK, CSF protein levels, and CSF) were calculated. The group of patients was divided into subgroups based on the disease duration (≤5 years/>5 years), body weight (≤80 kg/> 80 kg), INCAT total (0–4/5–10), the presence of diabetes mellitus type 2 (yes/no), hypertension (yes/no), CK level (normal/above normal), CSF pleocytosis (<5/>5 cells/*μ*l), protein (<50 g/dl/≥50 g/dl), and IgIV treatment (yes/no).

### 2.3. Electroneurography

The electrophysiological tests were carried out using the Viking Quest version 10.0 device. Motor and sensory nerves were performed using standard methods [25], with the evaluation of latency, amplitude, and conduction velocity. All tests were done under the same conditions. The same distance from the stimulating and active electrodes was used. The room temperature fluctuated between 21 and 23°C, and the temperature of the extremities was not less than 32°C. Compound muscle action potential (CMAP) was determined in the median, ulnar, peroneal, and tibial nerves. F-wave latency was studied for all motor nerves. Sensory nerve action potential (SNAP) was determined in the median, ulnar, and sural nerves.

### 2.4. Serum Collection and RNA Isolation

Blood samples were collected from CIDP patients and controls. Serum fractions were isolated within 2 hours after blood collection using 10-minute centrifugation at 1900 × g and then at 16,000 × g. For serum miRNA isolation, the miRNeasy Serum/Plasma Advanced Kit (Qiagen, Germany) was used according to the manufacturer's protocol. Then, miRNA samples were frozen and stored at -80°C.

### 2.5. Reverse Transcription

Reverse transcription reactions (RT) were performed using 2,5 *μ*l of extracted microRNAs, TaqMan microRNA Reverse Transcription Kit (Thermo Fisher Scientific, Foster City, CA, USA), and specific RT primers for miR-31-5p in a final volume of 7.5 *μ*l according to manufacturer's recommendation. All RT products were diluted twice with water.

### 2.6. ddPCR

Droplet digital PCR was applied as a method of miR-31expression analysis due to its high accuracy, reproducibility, and sensitivity [26]. The ddPCR reaction mixtures contained the following: 1.33 *μ*l of RT product, 1 *μ*l of TaqMan miRNA-specific probe (ID 002279, Thermo Fisher Scientific), 7.67 *μ*l of molecular biology-grade water, and 10 *μ*l of 2x ddPCR™ Master Mix for Probes (Bio-Rad). A total of 20 *μ*l of the reaction mixtures was loaded into a plastic cartridge with 70 *μ*l of Droplet Generation Oil for Probes in the QX200 Droplet Generator (all from Bio-Rad). The droplets obtained from each sample were then transferred to a 96-well PCR plate (Eppendorf, Hamburg, Germany). PCR amplifications were carried out in the C1000 Touch Thermal Cycler at 95°C for 10 min, followed by 40 cycles at 95°C for 3 sec and 60°C for 1 min and 1 cycle at 98°C for 10 min ending at room temperature (RT). Finally, the plate was loaded on a Droplet Reader (BioRad) and read automatically. Absolute quantification (AQ) of each miRNA was calculated from the number of positive counts per panel using Poisson distribution. The quantification of the target miRNAs is presented as the number of copies/100 *μ*l of serum.

### 2.7. Data Analysis

Statistical analyses were performed using Statistica 13.0 software. Normality of distributions was tested using the Shapiro-Wilk test. Due to the lack of normality of distribution for many variables, Spearman's rank correlation coefficient was used to analyze relationships between variables. Due to the same reason, subgroup (based on the disease duration, body weight, INCAT total, etc.) comparisons were made using the Mann–Whitney *U*. The level of statistical significance for all variables was set at alpha = 0.05.

## 3. Results

### 3.1. The Clinical and Electrophysiological Data of the Study Group

**Figure 1:**
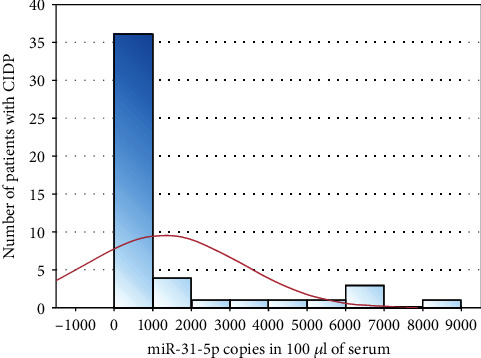
Histogram of miR-31-5p copies in 100 *μ*l of serum in the patient group.

The mean duration of CIDP was 5.31 years (SD ± 3.16). A total INCAT score was equal to 3.10 ± 2.14. The INCAT score for the upper limbs was 1.65 ± 0.98, and for the lower limbs, 1.77 ± 1.08. The mean body weight was 88.35 ± 20.05 kg; 28 patients (female: 4, male: 24) had a body weight > 80 kg. Blood tests results were as described: CK 269.02 ± 194.88 IU/l, IgG level 10.81 ± 2.55 g/l, IgA 2.42 ± 1.03 g/l, and IgM 1.07 ± 0.77 g/l. CSF general examination showed a mean pleocytosis amounting to 3.70 ± 2.9 cells/*μ*l, and the protein level was 74.20 ± 34.71 mg/dl. The mean copy number of miR-31-5p in 100 *μ*l serum was 1288.64 ± 2001.02 (Figure 1) in the CIDP group of patients, while in the control group, 3743.09 ± 4026.90.

Seventeen (17) patients had diabetes mellitus, and all of them were men.  Table 1 shows the clinical characteristics of patients with and without diabetes. The mean electrophysiological parameters for subjects are shown in Table 2.

The electrophysiological parameters were also compared between patients with and without concomitant diabetes mellitus. A significantly longer latency of the sural nerve SNAP was found in the diabetic group (4.25 ± 0.6 vs. 3.71 ± 0.86; *p* = 0.037). There was no statistical significance between the other parameters in the above groups.

### 3.2. The Correlations between MicroRNA-31-5p Copy Number and the Data Studied

**Figure 2:**
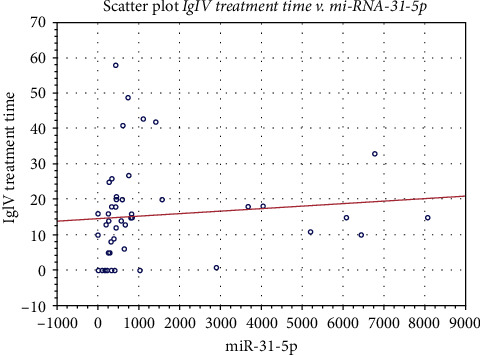
Scatter plot of the correlation between miR-31-5p expression and IgIV treatment time in CIDP patients.

A significant correlation of miR-31-5p was noted for the duration of IgIV treatment (0.426) (Table 3, Figure 2). Based on J. Guilford's classification, we showed a low correlation (0.1 < ∣*r* | ≤0.3) between microRNA-31-5p levels and IgG (0.212) and IgA (-0.209) levels, peroneal CMAP latency (0.25), tibial CMAP latency (0.24), and SNAP conduction velocity of the ulnar nerve (0.26). We did not find any significant correlation between microRNA-31-5p expression and other clinical, biochemical, and electrophysiological parameters (Tables 3 and 4).

The patients with body weight > 80 kg showed significantly lower levels of miRNA-31-5p than those with lower body weight (934.37 ± 1739.66 vs. 1784.62 ± 2271.62, respectively; *p* = 0.014). Conversely, patients with high protein levels in CSF showed significantly higher levels of miRNA-31-5p than those with normal protein levels (1393.93 ± 1932.27 vs. 987.38 ± 2364.10, respectively; *p* = 0.044).

### 3.3. The Group Characteristics in relation to IgIV Treatment

**Figure 3:**
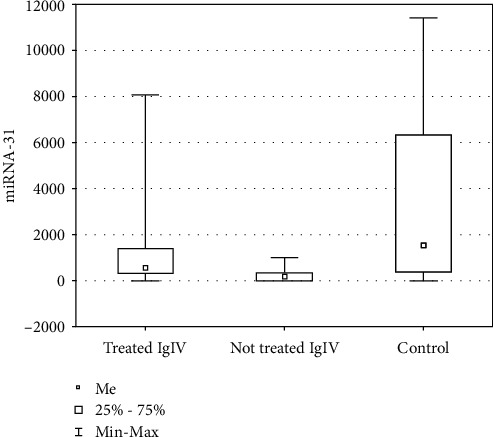
Graph showing the mean copy number of miR-31-5p in the patient group—treated and untreated with IgIV—and in the control group.

In the patient group, 38 subjects (female: 6, male: 32) were treated with IgIV. The patients who were not treated with IgIV demonstrated markedly decreased levels of miR-31-5p versus the patients who were treated (259.44 ± 304.02 vs. 1559.48 ± 2168.45, respectively; *p* = 0.002). The copy number of miR-31-5p in the group of patients treated with IgIV compared to the control group was lower but not statistically significant (1559.48 ± 2168.45 vs. 3743.09 ± 4026.90, respectively; *p* = 0.157), while in the group of patients not treated with IgIV compared to the control group, it was significantly lower (259.44 ± 304.02 vs. 3743.09 ± 4026.90, respectively; *p* = 0.014) (Figure 3).

In addition, the group of patients treated with IgIV showed significantly higher protein levels in the cerebrospinal fluid than the group of patients untreated with IgIV (79.59 ± 36.20 vs. 54.29 ± 18.90, respectively; *p* = 0.017). There were no significant statistical differences between IgA, IgG,and IgM levels between the groups of patients treated and untreated with IgIV.

### 3.4. The Characteristics of Patients with Upregulation of MicroRNA-31-5p Expression

Upregulation of microRNA-31-5p expression, determined as value > mean value + 3SD, was found in 5 patients. The mean copy number of miR-31-5p in 100 *μ*l serum was 6503.76. In this group of patients, the mean electrophysiological parameters were better than the mean values obtained in the entire CIDP group (Table 5). The biochemical parameters in this group of patients showed the following: CK 215.60 U/l, IgG level 9.3 g/l, IgA 2.7 g/l, and IgM 0.62 g/l. CSF general examination showed a mean pleocytosis amounting to 3.60 cell/*μ*l, and the protein level was 60.14 mg/dl. The mean duration of CIDP disease was 5 years, mean IgIV treatment time 16.80 months, and mean weight 96 kg. A total INCAT score was equal to 2.2, and the INCAT score for the upper limbs was 1.00 and for the lower limbs 1.40.

## 4. Discussion

The most important result of our study is the finding of low miRNA-31 values in CIDP patients. The small copies of microRNA-31 can be attempted to explain the reciprocal dysregulation of the transcription factors in patients with CIDP. Several studies demonstrate that miR-31 can positively regulate cell proliferation, differentiation, and activity by regulating NF-*κ*B, RAS/MAPK, Notch, and some cytokine signalling pathways [12, 14, 27, 28]. The increased miRNA-31 copies in our patients treated with IVIg may be due to the mechanism of action of IVIg. IVIg inactivates, silences, or leads to apoptosis of T cells, while restoring the balance of anti- and proinflammatory cytokines. Additionally, IVIg are thought to interfere with the passage of autoimmune cells across the blood-nerve barrier and to reduce antibody production by B cells, interfere with B cell proliferation through cell surface receptors, and block the activity of certain B cell subtypes. IVIg may also contain numerous anti-idiotypes that neutralise pathogenic antibodies. Finally, it has been shown that IVIg treatment interrupts several steps in the complement activation cascade and affects activity mediated by the Fc receptor [29–31].

The findings of Ripamonti et al. [32] highlighted miRNA-31 as a possible target for modulation of T cell-dependent antibody responses in humans with immune dysregulation. Antibody production by B lymphocytes requires support from follicular helper T (TFH) cells—a specific subgroup of CD4+ T lymphocytes. TFH cell role needs the presence of BCL6 (B cell lymphoma 6), a transcriptional repressor with unclear gene target responsible for helper activity. Combined miRNA analysis with gene expression profiling in human TFH cells, the authors found that the level of miR-31 is upregulated. The authors concluded that their findings highlight miR-31 as a possible target to modulate human T cell-dependent antibody responses in the settings of infection, vaccination, or immune dysregulation. In our study, we were able to confirm the upregulation process only in 5 patients. The patients with a high level of miRNA-31 had better clinical (in INCAT score) and electrophysiological parameters, although due to the small number of subjects, statistical analysis could not be performed. They also showed lower CK activity, lower IgG and IgM levels, as well as lower CSF protein levels.

We demonstrated a low positive correlation between miRNA-31 and serum IgG level. Previous studies have found that patients with Guillain-Barré syndrome (GBS) have high variability in serum immunoglobulin G (IgG) levels after standard IVIg treatment and that large increases in serum IgG (*Δ*IgG) are associated with better treatment outcome. A recent prospective study in CIDP indicated that the increased *Δ*IgG level after standard IVIg dosage in the continuous treatment was relatively constant within individual patients. This level could differ between patients who were treated with the same stable dosage and with interval of IVIg [33]. In our study, there were no statistically significant differences in IgG levels between treated and untreated IgIV groups, as well as in the patients with miRNA-31 upregulation. In the CIDP patients, we additionally revealed the low negative correlation between IgA and miRNA-31 levels, but no significant differences were found between IgA levels in the patient and healthy groups. In the course of IgA deficiency, the occurrence of autoimmune diseases has been found to be significantly more frequent than in patients with normal levels of this immunoglobulin. The relationships between IgA, IgG, and miRNA-31 in our study group were ambiguous and did not allow drawing clear conclusions.

We found a positive, average correlation between the duration of IVIg treatment and miRNA-31-5p expression. IVIg has been shown to inactivate or lead to T cell apoptosis, while restoring the balance of anti- and proinflammatory cytokines. Additionally, IVIg is thought to interfere with the passage of autoimmune cells across the blood-nerve barrier and to reduce antibody production by B cells, interfere with B cell proliferation through cell surface receptors, block the activity of certain B cell subtypes, interrupt several steps in the complement activation cascade, and affect activity mediated by the Fc receptor [31, 34]. The correlations between IVIg treatment effectiveness and miRNA level were described in many studies in different clinical situations [35–38]. IVIg replacement therapy in different immunodeficiency patients is thought to be able to modulate miRNA level. miRNAs seem to be a valid target to develop new therapies and potential biomarkers in different inflammatory diseases. Based on the literature and their original results, the authors proposed an additional hypothesis for the mechanism of miR-31-5p regulation [39–42]. According to the miRDB database, the target of miR-31-5p is the miRNA for the sphingomyelin synthase 1 (SGMS1) gene. Sphingomyelin synthase converts ceramide to sphingomyelin, so the amount of CSF sphingomyelin directly depends on its amount and activity. If in CIDP the level of miR-31-5p decreases (as we observe in our studies, CIDP patients not treated with IgIV have very low levels of this miR compared to control and treated patients), this may directly affect the increase in the level of its target, SGMS1, and consequently sphingomyelin. The application of IgIV therapy improves the condition of patients by, among other things, increasing miR31-5p (in our observations, patients after IgIV therapy have statistically higher levels of miR31-5p), which decreases SGM1A and consequently affects the reduction of sphingomyelin. On this basis, we can conclude that the level of circulating serum miR-31-5p may not only be a helpful diagnostic biomarker in CIDP but also a tracer of clinical improvement in patients with CIDP undergoing IgIV therapy.

We tried to consider other factors influencing the miRNA level in our patients and the results of treatment. The lower levels of miRNA-31 were observed in the patients with high body weight. There are many controversies regarding the importance of obesity in the CIDP patients and its influence on the IVIg treatment. Pharmacokinetic difference in lean and obese patients at higher doses of IVIg were observed, but it seems to be small to influence the IVIg dosage. Sometimes, a very high IgG level is observed in obese patients treated with IVIg [43]. The CIDP patients with and without diabetes mellitus were similar in the clinical and electrophysiological tests; miRNA-31, IgG, Ig A, CK, and CSF protein levels did not differ either. Our results were similar to other studies in which the differences between patients with and without DM were not seen [44].

Influencing miRNA function may contribute to a better understanding of the CIDP pathogenesis and the selection of an effective type of treatment. It is worth noting that abnormal expression of individual microRNAs may result from changes in the genome or abnormalities in their biogenesis or may be related to epigenetic factors regulating gene expression [45–49]. The work of Vinci et al. [47] highlighted that not only structurally determined changes in miRNA expression levels but also their epigenetic regulation seemed to be important in the CIDP pathogenesis and may become future targets for the development of new therapies modulating miRNA expression through changes in the level of methylation of their genes. Additionally, the interaction between miR-31 and the NF-*κ*B signalling pathway is the subject of many studies. The positive feedback loop formed by miR-31 and NF-*κ*B signalling may bring new ideas for the treatment of some autoimmune conditions [14, 48–52]. In a previous study, the authors demonstrated significantly higher levels of IL-6, IL-2, IL-4, and TNF-*α* and an increased CD4+/CD8+ ratio in patients with CIDP compared to controls. Electrophysiological parameters in CIDP patients were also found to be closely related to the autoimmune process. All these studies may contribute to a better understanding of the pathomechanism of CIDP [53].

The authors are aware of the study's limitations. Due to the complexity of miRNA roles, the main limitation was the selection of only one miRNA type. The selection was based on the literature and the importance of miRNA-31 related to CIDP issues. Additionally, this study and the control group were rather small. The patient group was not homogeneous in terms of disease duration, type of treatment, and additional medical conditions.

## 5. Conclusion

In this study, we have shown the reduced level of miRNA-31-5p in the patients with typical CIDP. It was significantly reduced in the patients who were not treated with IVIg. In the treated group, miRNA was approaching the correct level, and in some patients, it was upregulated.

These patients had better clinical and electrophysiological results than other patients with CIDP. We observed a clearly negative impact of the obesity on the clinical and electrophysiological status with a lower miRNA-31 level. All these results could support the thesis that miRNA-31 is highly involved in the autoimmune process in CIDP. The positive relationship between the higher level of miRNA-31 and the duration of IVIg treatment may be an additional factor explaining the effectiveness of the prolonged IVIg therapy in CIDP. The presence of diabetes mellitus does not significantly influence the miRNA-31 level and clinical and electrophysiological status. Further studies are needed to assess whether such miRNAs could represent novel potential biomarkers in the management and therapy of CIDP patients.

## Figures and Tables

**Table 1:** The clinical characteristics of patients without and with diabetes.

		Group of patients without diabetes		Group of patients with diabetes		*p* value
		Mean	SD	Mean	SD	
miR-31-5p/100 *μ*l of serum		1366.33	2054.54	1146.97	1953.03	0.332
Duration of the CIDP (years)		5.35	3.07	5.24	3.42	0.688
Duration of IgIV treatment (months)		17.16	13.01	12.00	14.90	0.076
Body weight (kg)		87.32	20.27	90.24	20.12	0.726
INCAT upper limbs		1.94	1.00	1.12	0.70	0.005
INCAT lower limbs		1.97	1.17	1.41	0.80	0.086
Serum	CK (IU/l)	279.13	197.57	250.59	194.48	0.698
Cerebrospinal fluid	IgG (g/l)	10.95	2.87	10.55	1.86	0846
	IgA (g/l)	2.29	0.86	2.65	1.28	0.532
	IgM (g/l)	1.18	0.83	0.87	0.63	0.200
	Pleocytosis (cells/*μ*l)	3.43	2.49	4.18	3.70	0.796
	Protein (mg/dl)	72.39	37.20	7741	30.66	0.499
Age (years)		59.23	11.93	65.94	10.40	0.070
*ni*			31		17	
Gender		F = 8	M = 23	F = 0	M = 17	

Abbreviation: *ni*: number of patients in the group.

**Table 2:** The mean electrophysiological parameters of CIDP patients.

Nerve conduction		Electrophysiological parameters	Patients *n* = 48
Motor	Median (wrist-elbow)	Latency (ms)	6.20 ± 3.94
		Amplitude (mV)	5.28 ± 3.75
		CV (m/s)	37.50 ± 18.26
	Ulnar (wrist-elbow)	Latency (ms)	3.60 ± 1.40
		Amplitude (mV)	7.04 ± 3.20
		CV (m/s)	46.48 ± 11.35
	Peroneal (ankle head of fibula)	Latency (ms)	6.16 ± 1.88
		Amplitude (mV)	2.01 ± 1.67
		CV (m/s)	37.19 ± 6.81
	Tibial (ankle popliteal fossa)	Latency (ms)	6.26 ± 1.38
		Amplitude (mV)	3.19 ± 3.13
		CV (m/s)	35.97 ± 7.74

Sensory	Median (digit II)	Latency (ms)	3.64 ± 1.03
		Amplitude (*μ*V)	11.01 ± 7.25
		CV (m/s)	42.08 ± 7.69
	Ulnar (digit V)	Latency (ms)	3.13 ± 0.94
		Amplitude (*μ*V)	10.36 ± 6.69
		CV (m/s)	42.96 ± 8.89
	Sural	Latency (ms)	3.82 ± 0.67
		Amplitude (*μ*V)	5.10 ± 3.92
		CV (m/s)	43.47 ± 9.85

F-wave studies	Median	F-latency (ms)	34.34 ± 6.95
	Ulnar		35.01 ± 7.74
	Peroneal		67.17 ± 8.25
	Tibial		67.62 ± 8.05

**Table 3:** Correlations of miR-31-5p with clinical and biochemical data in CIDP patients.

Data			Correlations
Clinical Parameters	Disease duration		-0.024
	IgIV treatment time		0.426
	Weight		-0.117
	INCAT	Upper limb	0.186
		Lower limb	0.109
		Total	0.167
Biochemical parameters	Serum	CK	-0.019
		IgG	0.212
		IgA	-0.209
		IgM	0.067
	Cerebrospinal fluid	Protein levels	0.123
		Pleocytosis	0.023

**Table 4:** Correlation between the electrophysiological parameters and expression of miRNA31-5p in serum in CIDP patients.

Nerve conduction		Electrophysiological parameters	Correlation miRNA
Motor	Median (wrist-elbow)	Latency	0.05
		Amplitude	-0.12
		CV	-0.08
	Ulnar (wrist-elbow)	Latency	0.02
		Amplitude	-0.04
		CV	-0.13
	Peroneal (ankle head of fibula)	Latency	0.25
		Amplitude	0.12
		CV	-0.01
	Tibial (ankle popliteal fossa)	Latency	0.24
		Amplitude	-0.05
		CV	0.12
Sensory	Median (digit II)	Latency	0.09
		Amplitude	-0.11
		CV	-0.01
	Ulnar (digit V)	Latency	-0.14
		Amplitude	-0.15
		CV	0.26
	Sural	Latency	-0.13
		Amplitude	-0.16
		CV	0.08
F-wave studies	Median	F-latency	-0.08
	Ulnar		0.09
	Peroneal		-0.09
	Tibial		0.01

**Table 5:** The mean electrophysiological parameters of CIDP patients with upregulation of microRNA-31 expression.

Nerve conduction		Electrophysiological parameters	Patients *n* = 5
Motor	Median (wrist-elbow)	Latency (ms)	6.38
		Amplitude (mV)	7.08
		CV (m/s)	47.4
	Ulnar (wrist-elbow)	Latency (ms)	2.96
		Amplitude (mV)	8.78
		CV (m/s)	47.2
	Peroneal (ankle head of fibula)	Latency (ms)	6.28
		Amplitude (mV)	2.12
		CV (m/s)	40.8
	Tibial (ankle popliteal fossa)	Latency (ms)	6.68
		Amplitude (mV)	3.30
		CV (m/s)	38.10
Sensory	Median (digit II)	Latency (ms)	3.52
		Amplitude (*μ*V)	11.30
		CV (m/s)	48.80
	Ulnar (digit V)	Latency (ms)	2.72
		Amplitude (*μ*V)	11.90
		CV (m/s)	44.60
	Sural	Latency (ms)	3.61
		Amplitude (*μ*V)	4.20
		CV (m/s)	44.10
F-wave studies	Median	F-latency (ms)	32.76
	Ulnar		32.48
	Peroneal		68.89
	Tibial		65.76

## Data Availability

The data sets used and/or analyzed during the current study are available from the corresponding author on reasonable request.

## Abbreviations

CSF: Cerebrospinal fluid
CIDP: Chronic inflammatory demyelinating polyneuropathy
CK: Creatine kinase
EAE: Experimental autoimmune encephalomyelitis
FOXP3: Forkhead box P3
GBSl: Guillain-Barré syndrome
IgIV treatment: Immunoglobulin intravenous treatment
miRNAs: MicroRNAs
miRNA-31-5p: MicroRNA-31-5p
Nuclear factor kappa B: Nuclear factor kappa B
SGMS1: Sphingomyelin synthase 1.
